# Anomalous Thermal Expansion of HoCo_0.5_Cr_0.5_O_3_ Probed by X-ray Synchrotron Powder Diffraction

**DOI:** 10.1186/s11671-017-2213-7

**Published:** 2017-07-05

**Authors:** Vasyl Hreb, Leonid Vasylechko, Vitaliya Mykhalichko, Yurii Prots

**Affiliations:** 1 Lviv Polytechnic National University, 12 Bandera Street, 79013 Lviv, Ukraine; 2 Max-Planck-Institut für Chemische Physik fester Stoffe, Nöthnitzer Str. 40, 01187 Dresden, Germany

**Keywords:** Mixed cobaltites-chromites, Perovskite, Crystal structure, Thermal expansion

## Abstract

Mixed holmium cobaltite-chromite HoCo_0.5_Cr_0.5_O_3_ with orthorhombic perovskite structure (structure type GdFeO_3_, space group *Pbnm*) was obtained by solid state reaction of corresponding oxides in air at 1373 K. Room- and high-temperature structural parameters were derived from high-resolution X-ray synchrotron powder diffraction data collected in situ in the temperature range of 300–1140 K. Analysis of the results obtained revealed anomalous thermal expansion of HoCo_0.5_Cr_0.5_O_3_, which is reflected in a sigmoidal temperature dependence of the unit cell parameters and in abnormal increase of the thermal expansion coefficients with a broad maxima near 900 K. Pronounced anomalies are also observed for interatomic distances and angles within Co/CrO_6_ octahedra, tilt angles of octahedra and atomic displacement parameters. The observed anomalies are associated with the changes of spin state of Co^3+^ ions and insulator-metal transition occurring in HoCo_0.5_Cr_0.5_O_3_.

## Background

Rare earth (*R*) cobaltites *R*CoO_3_ and chromites *R*CrO_3_ with perovskite structure due to their high electrical conductivity, specific magnetic properties, as well as significant electrochemical and catalytic activity are considered as prospective electrode and interconnect materials for solid oxide fuel cells (SOFC) [1–3], thermoelectric and magnetocaloric materials [4–6], catalysts and humidity and gas sensors [7–9]. Currently *R*CoO_3_ and *R*CrO_3_ compounds and solid solutions on their basis are attracting renewed research interest aroused by their potential application as multifunctional materials [10–13]. *R*CoO_3_-based materials are of particular interest, due to dependency of their transport, magnetic and other properties on spin state of Co^3+^ ions, which can change with increasing of the temperature from low spin (LS, *t*_2*g*_^6^ *e*_*g*_^0^, *S* = 0), to intermediate (IS, *t*_2*g*_^5^ *e*_*g*_^1^, *S* = 1) and high spin (HS, *t*_2*g*_^4^ *e*_*g*_^2^, *S* = 2) configurations ([14–16] and references herein). These transitions in rare earth cobaltites *R*CoO_3_ are strongly affected by the chemical pressure caused by cation substitution either in *A*- or *B*-sites of perovskite structure [17–19].

The present work deals with the study of crystal structure of new mixed cobaltite-chromite HoCo_0.5_Cr_0.5_O_3_ and its thermal behaviour in the temperature range of 300–1140 K by using high-resolution X-ray synchrotron powder diffraction technique. The HoCo_0.5_Cr_0.5_O_3_ was chosen for the detail structural investigations as a representative of the mixed cobaltites-chromites in view of the fact, that both parent compounds—HoCoO_3_ and HoCrO_3_, which are isostructural and isotypic with GdFeO_3_ [20–23], show a variety of intriguing physical phenomena and properties. In particular, holmium chromite undergoes a low-temperature phase transition from centrosymmetric *Pbnm* to the non-centrosymmetric *Pna*2_1_ structure, as it was recently suggested by X-ray powder diffraction of HoCrO_3_ at 80 and 160 K [12]. The authors assume that the polar oxygen rotations of CrO_6_ octahedra combined with the displacements of Ho in the non-centrosymmetric space group *Pna*2_1_ engineer ferroelectricity in HoCrO_3_ below 240 K. For HoCoO_3_ no structural phase transitions are reported in a broad temperature range between 1.5 and 1098 K, although pronounced anomalies are observed both in low- and high-temperature lattice expansion [24–26]. A negative expansion observed in *b*-direction (in *Pbnm* setting) below 150 K suggests a magnetoelastic coupling where short-range interactions between Ho^3+^ magnetic moments are established [24]. The high-temperature anomalies are associated with the transitions of the Co^3+^ ions to the higher spin states and coupled metal-insulator transition occurred in HoCoO_3_ above 780 K [15, 25, 26]. On the assumption of aforesaid extremely complicated structure, magnetic and electronic phase behaviour is expected in the mixed cobaltite-chromite system HoCo_0.5_Cr_0.5_O_3_. Analysis of the thermal expansion behaviour is a very useful tool for the investigation of diverse electronic and magnetic phase transformations occurring in the complex oxide perovskite systems [14, 16, 19].

## Methods

HoCo_0.5_Cr_0.5_O_3_ was synthesized by a solid state technique. Precursor oxides Ho_2_O_3_, Co_3_O_4_ and Cr_2_O_3_ were ball-milled in ethanol for 5 h, dried, pressed into pellet and annealed in air at 1373 K for 20 h. After regrinding, the product was repeatedly ball-milled in ethanol for 2 h, dried and annealed in air at 1373 K for 45 h with one intermediate regrinding.

X-ray powder diffraction (Huber imaging plate Guinier camera G670, Cu *K*_α1_ radiation) was used for the characterization of the sample at room temperature. Thermal behaviour of HoCo_0.5_Cr_0.5_O_3_ crystal structure was studied in situ in the temperature ranges of 300–1140 K by using high-resolution X-ray synchrotron powder diffraction (beamline ID22 at ESRF, Grenoble, France). The data were collected upon the heating of the powdered sample filled into 0.3 mm quartz capillary with the temperature step of 50 K. The wavelength used *λ =* 0.35434 Å allows to collect the diffraction data until the maximum sinΘ/λ value of 0.849 ensuring reliable information on the positional and displacement parameters of atoms in HoCo_0.5_Cr_0.5_O_3_ structure at the elevated temperatures. Corresponding structural parameters were derived by full-profile Rietveld method implemented in the program package WinCSD [27].

## Results and Discussion

**Fig. 1:**
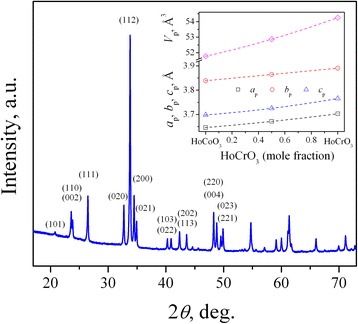
XRD pattern of HoCo_0.5_Cr_0.5_O_3_ at room temperature (Cu *K*_α1_ radiation, Guinier camera). Insets show concentration dependence of the unit cell parameters in the HoCoO_3_–HCrO_3_ system. The orthorhombic lattice parameters are normalized to the perovskite cell as follows: *a*_p_ = *a*_o_/√2, *b*_p_ = *b*_o_/√2, *c*_p_ = *c*_o_/2, *V*_p_ = *V*_o_/4

X-ray powder diffraction examination of new mixed cobaltite-chromite HoCo_0.5_Cr_0.5_O_3_ revealed almost pure perovskite structure isotopic with GdFeO_3_ (Fig. 1). The obtained values of unit cell dimensions are in excellent agreement with the corresponding data for the parent HoCoO_3_ and HoCrO_3_ compounds (Fig. 1, inset 1), thus proving an apparent formation of continuous solid solution HoCo_1–*x*_Cr_*x*_O_3_ with perovskite structure, similarly to the related *R*CoO_3_–*R*CrO_3_ systems with La, Pr, Nd, Sm, Eu, Gd, Dy, Er and Y [18, 19, 28–33].

**Fig. 2:**
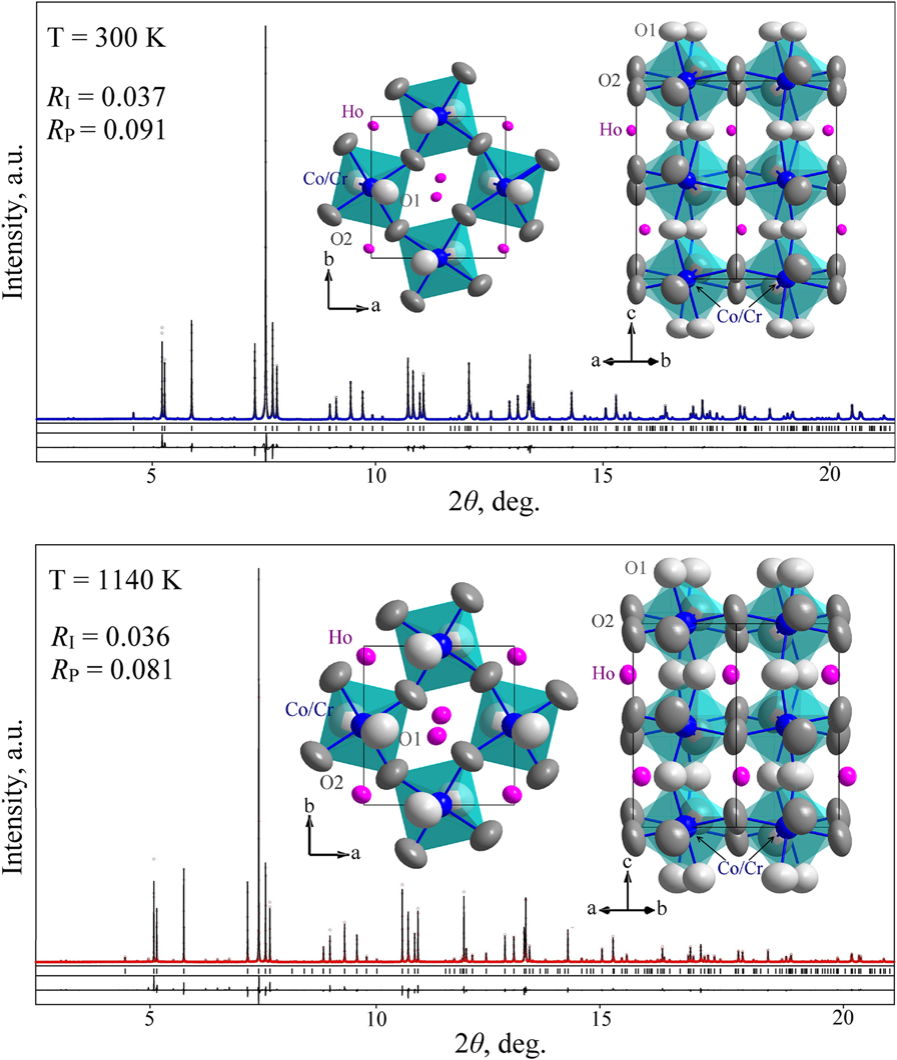
X-ray synchrotron powder diffraction patterns of HoCo_0.5_Cr_0.5_O_3_ at 300 and 1140 K. Experimental (*dots*) and calculated patterns, difference profiles and positions of the diffraction maxima are given. Insets show corresponding structures in projections on (001) and (110) planes. The displacement ellipsoids of atoms are shown at 90% probability level

In situ high-temperature X-ray synchrotron powder diffraction revealed that HoCo_0.5_Cr_0.5_O_3_ remains orthorhombic up to highest investigated temperature of 1140 K. No symmetry-related structural changes were observed. Precise crystal structure parameters of HoCo_0.5_Cr_0.5_O_3_ in the temperature range of 300–1140 K including anisotropic displacement parameters for all atomic positions were derived by full-profile Rietveld refinement. In all cases, the refinement procedure performed in space group *Pbnm* led to the excellent agreement between experimental and calculated profiles. Selected examples of Rietveld refinement at 300 and 1140 K are presented on Fig. 2. Insets on Fig. 2 show corresponding projections of HoCo_0.5_Cr_0.5_O_3_ structure on (001) and (110) planes with thermal ellipsoids of atoms based on the refined structural parameters presented in Table 1.

**Table 1:** Coordinates and atomic displacement parameters in HoCo_0.5_Cr_0.5_O_3_ structure (space group *Pbnm*) at 300 and 1140 K

Parameters	Ho, 4*c*		Co/Cr, 4*b*		O1, 4*c*		O2, 8*d*	
	300 K	1140 K	300 K	1140 K	300 K	1140 K	300 K	1140 K
*x/a*	−0.01617(9)	−0.0155(2)	0		0.1043(9)	0.1074(11)	−0.3090(7)	−0.3130(9)
*y/b*	0.06694(8)	0.0654(1)	½		0.4670(9)	0.4675(12)	0.3057(7)	0.3061(9)
*z/c*	¼		0		¼		0.0541(5)	0.0539(6)
*B*_eq_^a^	0.556(8)	1.52(2)	1.65(3)	2.63(5)	2.9(2)	5.0(3)	3.14(14)	4.7(2)
*B*_11_	0.61(2)	1.53(3)	1.64(5)	2.68(8)	3.6(3)	5.7(5)	2.9(2)	4.2(3)
*B*_22_	0.48(2)	1.33(2)	1.74(5)	2.64(9)	3.2(3)	5.2(5)	2.5(2)	3.8(3)
*B*_33_	0.58(2)	1.72(3)	1.56(5)	2.57(7)	1.9(3)	4.2(4)	4.0(3)	6.1(4)
*B*_12_	−0.07(2)	−0.13(4)	0.05(6)	−0.03(10)	0.2(3)	0.6(4)	−1.0(2)	−1.4(3)
*B*_13_	0		−0.04(6)	−0.04(10)	0		0.5(2)	−0.2(3)
*B*_23_	0		−0.02(4)	0.03(5)	0		−0.3(2)	−0.8(3)
Lattice parameters	*a* = 5.19695(8) Å, *b* = 5.46911(8) Å, *c* = 7.45765(9) Å (at 300 K)							
	*a* = 5.28195(7) Å, *b* = 5.55944(7) Å, *c* = 7.56949(9) Å (at 1140 K)							

^a^ *B*_iso/eq_ = 1/3[*B*_11_(a*)^*2*^ *a* ^2^ *+ …* 2*B*_23_ b*c*bc cos*α*]; displacement factors are defined as *exp*[−1/4(*B*_11_ (a*)^*2*^ *h* ^2^ *+ …* 2*B*_*23*_ b*c*k l)]

Crystal structure of HoCo_0.5_Cr_0.5_O_3_ is visualized as 3D framework of corner-shared *M*O_6_ octahedra (*M* = Co_0.5_Cr_0.5_) with the Ho atoms occupying hollows between them. The *M*O_6_ octahedra are rather distorted due to displacement of oxygen atoms from their “ideal” positions in the cubic perovskite aristotype. Mutual displacements of oxygen atoms are reflected in the cooperative antiphase tilts of *M*O_6_ octahedra, as is depicted on insets of Fig. 2.

The ratio of the atomic displacement parameters (adps) observed in HoCo_0.5_Cr_0.5_O_3_ structure both at 300 and 1140 K follow well the simple expectation based on the atomic masses, namely *B*_iso/eq_(O) > *B*_iso/eq_(Co/Cr) > *B*_iso/eq_(Ho). Thermal ellipsoids of cations in HoCo_0.5_Cr_0.5_O_3_ structure at room temperature are close to spherical shape, with minor contraction or elongation in *b*-direction: *B*_11_ *≈ B*_33_ *> B*_22_ for Ho^3+^ and *B*_11_ *≈ B*_33_ *< B*_22_ for Co^3+^/Cr^3+^. More pronounced anisotropic behaviour is observed for the displacement parameters of oxygen species, reflected in the remarkable contraction or elongation of the corresponding ellipsoids in *c*-direction (Table 1). Thermal ellipsoids of oxygen atoms both in equatorial (8*d*) and apical (4*c*) positions of *M*O_6_ octahedra show near rotation-type behaviour along *M*–O bonds (Fig. 2, insets). At the elevated temperatures, the displacement ellipsoids for Co/Cr atoms become almost spherical, whereas those for Ho^3+^ species exhibit considerable anisotropy, e.g. *B*_33_ *> B*_11_ *> B*_22_ at 1140 K. The behaviour of adps of oxygen species located in 4*c* and 8*d* sites (*B*_11_ *≈ B*_22_ *> B*_33_ and *B*_11_ *≈ B*_22_ *< B*_33_, respectively) does not change with the temperature (Table 1). However, it can be noticed that displacement parameters of apical O1 atoms located in 4*c* sites become more isotropic at the elevated temperatures (Fig. 2, insets).

**Fig. 3:**
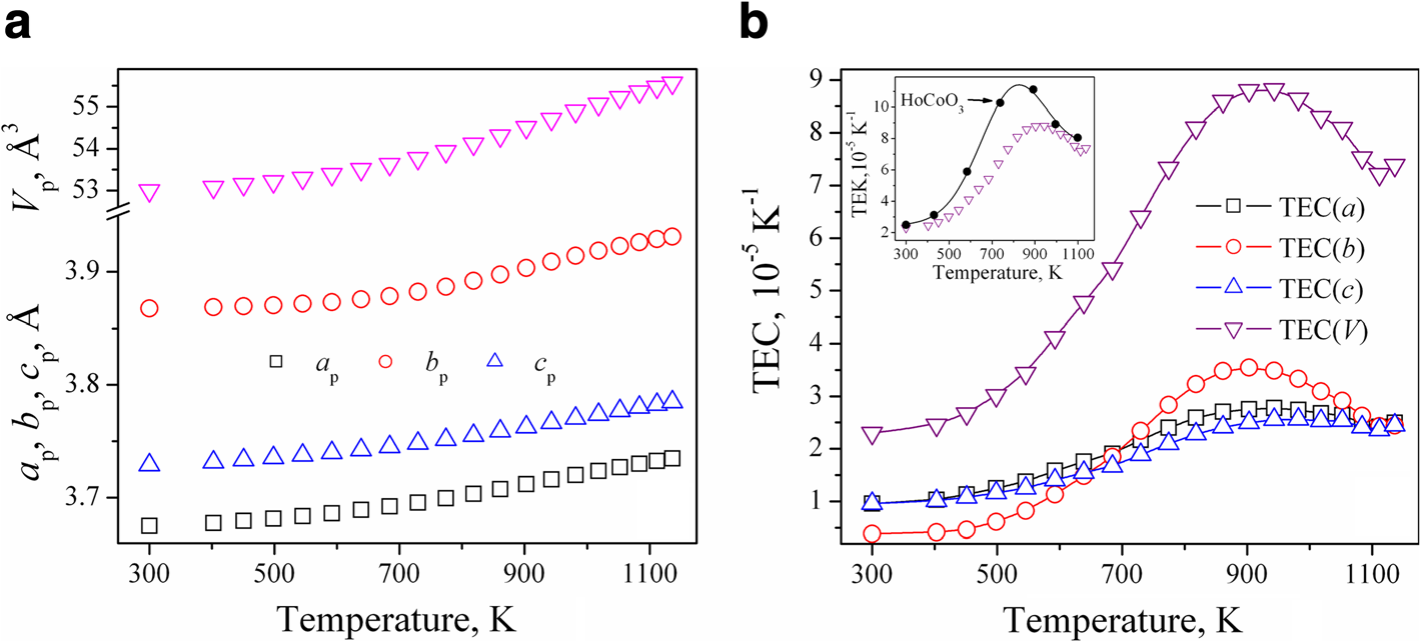
Temperature evolution of the normalized unit cell parameters (**a**) and linear thermal expansion coefficients (**b**) of HoCo_0.5_Cr_0.5_O_3_. The orthorhombic lattice parameters are normalized to the perovskite cell as follows: *a*_p_ = *a*_o_/√2, *b*_p_ = *b*_o_/√2, *c*_p_ = *c*_o_/2, *V*_p_ = *V*_o_/4. The values on linear TECs in three crystallographic directions as well volumetric TEC were obtained by differentiation of experimental unit cell dimensions on the temperature. Inset on the *right panel* shows volumetric TEC of HoCo_0.5_Cr_0.5_O_3_ in comparison with literature data for HoCoO_3_ [25]

Analysis of the thermal behaviour of HoCo_0.5_Cr_0.5_O_3_ structure revealed pronounced anomalies in the lattice expansion, which are reflected in a sigmoidal temperature dependence of the unit cell dimensions and in significant increase of the thermal expansion coefficients (TECs) with broad maxima around 900 K (Fig. 3). Similar abnormal lattice parameter behaviour was earlier observed in the related mixed cobaltites-chromites LaCo_1–*x*_Cr_*x*_O_3_ [28] and *R*Co_0.5_Cr_0.5_O_3_ (*R* = Pr, Sm, Eu, Gd, Dy and Er) [19, 31–33].

In the “pure” rare earth cobaltites *R*CoO_3_ abnormal thermal behaviour of the lattice expansion is associated with magnetic phase transitions and with a change of electronic configuration and spin state of Co^3+^ ions, which lead to the increment of the lattice parameters and unit cell volume due to increase of the radii of Co^3+^ ions in the exited states (*r*(LS) = 0.545 Å, *r*(IS) = 0.560 Å, *r*(HS) = 0.610 Å). The maxima at the temperature dependence of the thermal expansion coefficients in rare earth cobaltites show clear correlation with the temperature of insulator–metal transition, obtained from resistivity measurements, which increases in the *R*CoO_3_ series from 535 K for LaCoO_3_ to 785 and 800 K for DyCoO_3_ and YCoO_3_, respectively [14].

It is assumed that the observed structural anomalies in HoCo_0.5_Cr_0.5_O_3_ around 900 K are also associated with the magnetic and electronic phase transitions occurred at the elevated temperatures in the end members of this system. In particular, according to the electronic phase diagram of the *R*CoO_3_ perovskites [15], HoCoO_3_ undergoes a transition from nonmagnetic dielectric to paramagnetic dielectric state at 486 K and insulator–metal transition at 782 K. Detected anomalies in the lattice expansion of the mixed cobaltite-chromite HoCo_0.5_Cr_0.5_O_3_ are less pronounced than in the “pure” HoCoO_3_ [25], whereas the maximum at TEC curve is shifted to the higher temperatures (inset of Fig. 3b). Similar effect of cationic exchange was observed in the related *R*CoO_3_–*R*CrO_3_ systems, where increasing chromium content in NdCo_1–*x*_Cr_*x*_O_3_ and GdCo_1–*x*_Cr_*x*_O_3_ series led to increase of the temperature of metal–isolator transitions [18, 30].

**Fig. 4:**
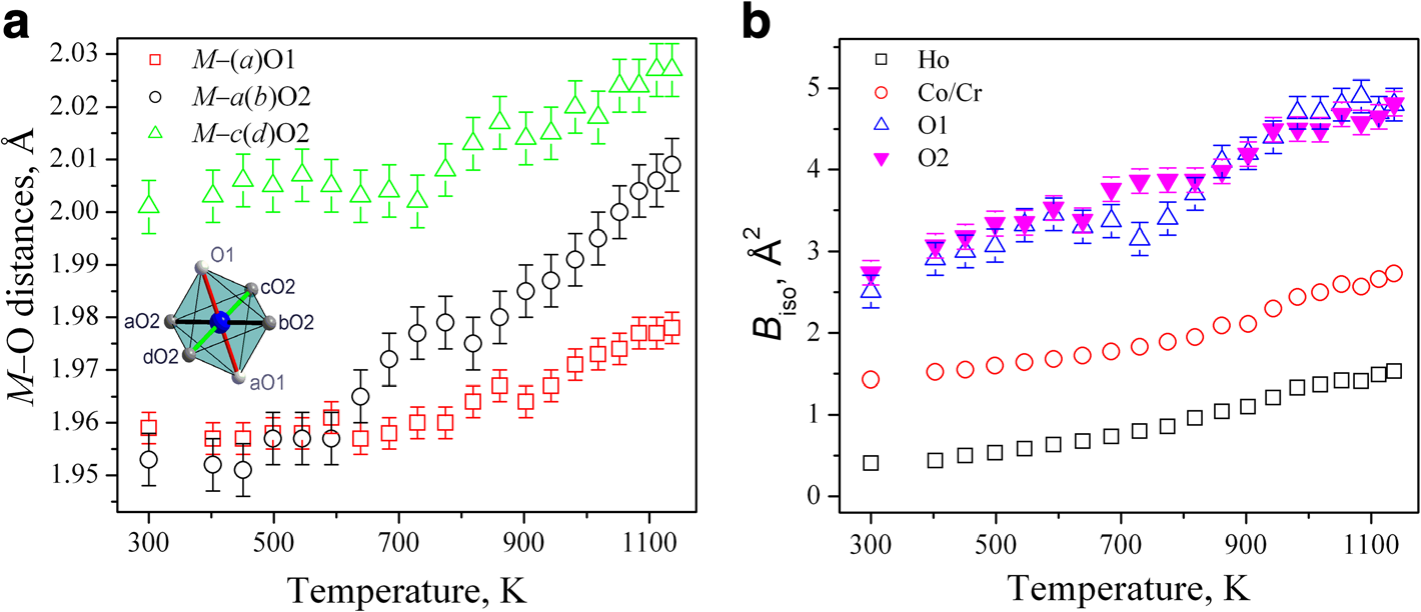
Temperature evolution of *M*–O bond lends (**a**) and isotropic displacement parameters of atoms (**b**) in HoCo_0.5_Cr_0.5_O_3_ structure

Thorough analysis of the selected bond length, atomic displacement parameters and octahedral tilt angles in HoCo_0.5_Cr_0.5_O_3_ structure indicates additional structural anomalies, which are evidently associated with the electronic and magnetic phase transitions occurring in the HoCoO_3_–HoCrO_3_ system at elevated temperatures. Temperature evolution of the *M*–O bond lengths in the HoCo_0.5_Cr_0.5_O_3_ structure is presented on Fig. 4a. Initially, both *M*–O1 and *M*–O2 distances remain practically unchanged. Significant change in configuration of *M*O_6_ octahedra occurs between ~600 and 850 K, where an excitation to the higher spin states of Co^3+^ ions begins. Detectable deviation from the “normal” behaviour in this temperature range is also observed for the temperature dependence of the displacement parameters of oxygen species in HoCo_0.5_Cr_0.5_O_3_ structure (Fig. 4b). Further increasing of the temperature led to the increase of all *M*–O distances and to the convergence of both sets of *M*–O2 bond lengths in the equatorial plane of *M*O_6_ octahedra (Fig. 4a). Thus, the shape of *M*O_6_ octahedra at the elevated temperatures differs considerably from the room temperature configuration.

**Fig. 5:**
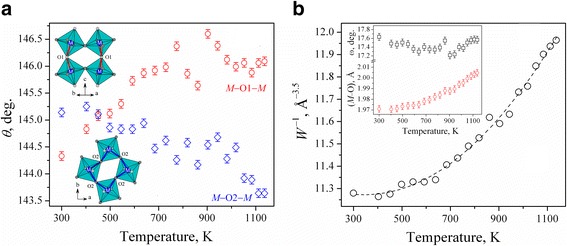
Temperature evolution of the *M*–O–*M* angles (**a**) and inverse bandwidth *W* ^−1^ (**b**) in HoCo_0.5_Cr_0.5_O_3_ structure. Inset shows thermal behaviour of the average bong length *M–*O and octahedral tilt angles

Temperature evolution of the *M*–O1–*M* and *M*–O2–*M* bond angles in HoCo_0.5_Cr_0.5_O_3_ structure reflecting the magnitude of *M*O_6_ octahedral tilt angles along [110] and [001] axis (Fig. 5a) displays clear divergence behaviour. The *M*–O2–*M* angles systematically decrease with increasing the temperature, whereas *M*–O1–*M* angles show increasing behaviour with detectable discontinuity between 770 and 900 K.

It is known that the *M*–O–*M* bong angles (*θ*) in *RM*O_3_ perovskite series characterize the *M* ^3+^–O^2−^–*M* ^3+^ overlaps and govern the magnetic and transport properties of rare earth manganites, nickelates and cobaltites [34, 35]. In particular, increase of cooperative rotations of corner-shared CoO_6_ octahedra in *R*CoO_3_ perovskites led to reducing of Co–O–Co bond angles and the bandwidth of Co(3*d*)–O(2*p*) interactions, which are correlated with the increasing spin-state transition temperature, *T*_onset_ [15]. According to ([15, 35] and references herein), in the *R*CoO_3_ cobaltite series the σ*-bonding *e*_g_ bandwidth *W* ∝ cos*ω*/〈Co–O〉^3.5^, where *ω* = (180 – 〈*θ*〉)/2 is the average octahedral tilting angle, and 〈Co–O〉—the mean bond length inside CoO_6_ octahedra. The broadening of *W* in rare earth cobaltite series reduces the spin gap and decreases the onset of spin transition of Co^3+^ from LS to IS state [15]. Figure 5b demonstrates the temperature dependence of the inverse bandwidth, *W* ^−1^ of HoCo_0.5_Cr_0.5_O_3_, which increase with the temperature solely due to increase of the average bond lengths inside octahedra, whereas the octahedral tilt angles are practically temperature independent (Fig. 5b, inset). Observed increasing behaviour of the inverse bandwidth of HoCo_0.5_Cr_0.5_O_3_ clearly illustrates an increasing population of the exited spin states of Co^3+^ ions with the temperature. It is apparent that the magnetic and electrical properties of HoCo_0.5_Cr_0.5_O_3_ will depend on the spin state of the Co^3+^ ions and a cation–anion–cation overlap, as it was reported for the related NdCo_1–*x*_Cr_*x*_O_3_ and GdCo_1–*x*_Cr_*x*_O_3_ systems [18, 30]. Increasing structural deformation in the last systems caused by the substitution of chromium by cobalt shifts the onset of Co^3+^ spin excitations and metal-insulator transition to the highest temperatures and led to the rising of electrical conductivity and Néel temperature in NdCo_1–*x*_Cr_*x*_O_3_ series. It is evident that the coupling of the electronic and magnetic transitions combined with the anomaly of the lattice behaviour will result in extremely complicated magnetic and electronic phase diagram of the mixed cobaltite-chromite systems.

## Conclusions

Crystal structure parameters of the mixed holmium cobaltite-chromite HoCo_0.5_Cr_0.5_O_3_ synthesized by solid state reaction in air at 1373 K have been studied in the temperature range of 300–1140 K by using high-resolution X-ray synchrotron powder diffraction technique. Experimental X-ray synchrotron powder diffraction patterns and crystal structure parameters of HoCo_0.5_Cr_0.5_O_3_ structure at room temperature and 1140 K are published by the International Centre of Diffraction Data (ICDD) in the last release of the Powder Diffraction File (PDF cards NN 00-066-0678 and 00-066-0679, respectively). Detailed analysis of the temperature dependence of structural parameters revealed pronounced anomalies in thermal behaviour of the unit cell dimensions and thermal expansion coefficients with clear maxima at around 900 K. Extra structural anomalies are also observed on temperature dependencies of the *M*–O bond lengths, octahedral tilt angles and atomic displacement parameters, which are evidently caused with the temperature induced changes of spin configuration of Co^3+^ ions and coupled metal-insulator transition occurred in HoCoO_3_–HoCrO_3_ system.
